# The protective and detrimental effects of self-construal on perceived rejection from heritage culture members

**DOI:** 10.3389/fpsyg.2015.00100

**Published:** 2015-02-16

**Authors:** Nelli Ferenczi, Tara C. Marshall, Kathrine Bejanyan

**Affiliations:** Department of Psychology, Brunel UniversityLondon, UK

**Keywords:** self-construal, intragroup marginalization, heritage culture, psychological adjustment, perceived rejection, social identity, independent self, interdependent self

## Abstract

Individuals may perceive themselves as interdependent and similar with close others, or as independent and distinct. Do these differences in self-construal influence perceptions of rejection from those closest to us? Few studies have investigated the antecedents of intragroup marginalization – the perception of rejection from family and friends due to not conforming to the prescribed values and expectations of one’s heritage culture. Furthermore, the implications of perceived intragroup marginalization for psychological adjustment and an integrated bicultural identity are unclear. To gage the effects of self-construals on perceived intragroup marginalization and psychological adjustment (i.e., subjective well-being and flourishing) and an integrated bicultural identity, we increased the cognitive accessibility of independent and interdependent self-construals through a priming manipulation. Participants were recruited via Amazon MTurk and completed the measures online. Our results showed that priming an interdependent self-construal decreased perceived intragroup marginalization from family and, in turn, poor psychological adjustment and bicultural identity conflict. Conversely, participants primed with an independent self-construal reported increased perceptions of intragroup marginalization from their family and, in turn, decreased psychological adjustment and increased identity conflict. These findings support the benefits of an interdependent self and the disadvantages of an independent self for minimizing perceived exclusion from heritage culture members.

“No man is an island, entire of itself; every man is a piece of the continent.”

John Donne

## INTRODUCTION

What is the fate of the individual who feels like an island, rather than safely anchored to land, amidst the cultural oceans? In this era of globalization, individuals often juggle the conflicting demands of multiple cultural identities (Castillo et al., 2007). Specifically, bicultural individuals may identify with the dominant culture in which they live, but feel pressured by members of their heritage culture to maintain a prescribed cultural identity. For example, a Latino American may feel criticized by other Latino Americans for not speaking Spanish fluently, or a British Asian may perceive rejection by other British Asians for acting “too British.” The antecedents of feeling like an accepted and valued member of one’s heritage culture and, in turn, the ramifications for one’s adjustment, have been overlooked in cross-cultural research. Intragroup marginalization refers to the experience of perceived rejection from heritage culture family and friends due to acculturating in ways deemed a threat to the normative values of the group’s social identity (Castillo et al., 2007; Thompson et al., 2010). What factors shape these perceptions of rejection? Notwithstanding the importance of self-construals for shaping our attitudes toward in-group members (Markus and Kitayama, 2010), no study until now has examined the role of self-construals in perceiving rejection from heritage culture members. Returning to Donne’s words, do we differentially perceive rejection depending on whether we construe ourselves as islands, separate from others, or, conversely, as inextricable parts of a continent?Previous research has focused on intragroup marginalization as a predictor of psychological adjustment (Castillo et al., 2008, 2012; Cano et al., 2014). Few studies, however, have examined the predictors of intragroup marginalization itself. Extending previous work, which showed that insecure attachment orientations are associated with increased intragroup marginalization (Ferenczi and Marshall, 2014), in this paper we examine independent and interdependent self-construals as additional predictors of perceiving intragroup marginalization. Viewing the self as unique (the independent self) or as similar to important others (the interdependent self) may influence perceived marginalization from in-group members. To this end, we primed participants with independent and interdependent self-schemata, which temporarily increases the cognitive accessibility of these representations and mimics the influence of chronic self-construals (Trafimow et al., 1991). In particular, this priming method increases or decreases perceptions of similarity with close others. Our study advances theory by being the first to investigate the link between self-construal and perceived intragroup marginalization. Furthermore, by examining the predictors of perceptions of intragroup marginalization, our study may have real-world implications for minimizing its negative effects on psychological adjustment and an integrated bicultural identity.

### SELF-CONSTRUAL

Independent self-construals are characterized by personal agency (Weisz et al., 1996; Imada and Ellsworth, 2011) and perceptions of a distinct, unique, and static inner self (Markus and Kitayama, 1991). They are prevalent within individualistic, Western, cultures, where it is valued to develop and attend to one’s inner attributes (e.g., motives, traits, and values) and personal goals (van Horen et al., 2008). Individuals rely on their inner self – which is perceived as being consistent (Suh, 2002) – to interpret and imbue behavior with meaning (Morris and Peng, 1994).These values are reflected in cultural institutions, such as the prevalence of narratives describing achievement and self-direction in American textbooks (Imada, 2010). Other individuals are still significant, but are cast into the roles of affirmers and appraisers, relied on to verify the inner self. The onus is on the individual to express their inner self if they wish to be understood.

Interdependent self-construals, conversely, are characterized by a focus on harmonious relationships, attending to others, and fitting into the in-group (Imada, 2010). They are prevalent in collectivistic, Asian, cultures. The interdependent self may behave in different ways across differing situations depending on what is deemed appropriate (Markus and Kitayama, 1991). Thus, core attributes of the self are situation-specific and can be dialectical or contradictory (Peng and Nisbett, 1999). In contrast to the independent self, the interdependent self directs control inward to ensure that private emotions do not displace the equilibrium of harmonious interpersonal interaction. Notably, interdependent individuals are more sensitive to disharmony, expressing more concern about potential relationship conflict (Bejanyan et al., 2014). Pro-relationship traits and caring behaviors form a stronger basis for their self-esteem than they do for independent selves (Goodwin et al., 2012). Because close others actively participate in the construction and definition of the self, the interdependent self is constantly aware of others’ needs, goals, and expectations. Self-esteem is contingent on fitting into the in-group and living up to their standards (Hannover et al., 2006). Significantly, the interdependent self is not indiscriminate; only in-group members are incorporated into the self. The significance of incorporating others in the interdependent self is evidenced in the representation of close family members in the same location as the self on a neural level (Ng et al., 2010). It is logical to surmise that the differing ways in which individuals construct their self-concept, in particular when conceptualizing the boundary between self and others, will influence their perceptions of rejection from close members of their heritage culture.

### INTRAGROUP MARGINALIZATION

Social rejection tends to be an extremely painful experience (MacDonald and Leary, 2005). It shares similar neural correlates with physical pain, supporting the significance of the social attachment system in an evolutionary context (Eisenberger et al., 2003). The Social Identity Theory approach posits that important social groups are incorporated into a distinct part of identity, known as social identity (Tajfel, 1974, 1982). Being a member of a group plays a significant role in psychological well-being. Thus, the experience of rejection from in-group members is particularly painful when bound up with the implication that one is reflecting poorly on a shared social identity (Haslam et al., 2009).

Non-conforming group members are punished more severely than out-group members as they may impair their group’s positive identity (the ‘Black Sheep’ effect; Marques and Yzerbyt, 1988; Marques et al., 1988). Indeed, individuals can come to perceive that they are the ‘black sheep’ of their heritage cultures. In this vein, they may experience intragroup marginalization – perceiving rejection from other heritage culture members because they adopt the values, behaviors, and norms of the mainstream culture in ways that are threatening to the heritage culture social identity (Castillo et al., 2007, 2008). Heritage culture refers to the culture of one’s birth or a culture that had a significant impact on previous generations of one’s family; the mainstream culture is the culture of current residence. At its core, intragroup marginalization is the confrontation of an individual with accusations of betrayal and ‘selling out’ from members of their heritage culture community (Castillo et al., 2007). Further support for intragroup marginalization is found in the exacerbated tendency for participants to rate in-group non-conformists unfavorably when they are aware of an out-group (Abrams et al., 2000). In intergroup contexts, such as societies where there is continuous awareness of multiple cultures, the punishment and resulting social rejection for not maintaining the norms of the heritage culture social identity may be severe.

### THE INFLUENCE OF SELF-CONSTRUAL ON INTRAGROUP MARGINALIZATION

We propose that an interdependent self-construal may be linked with decreased perceptions of intragroup marginalization. By emphasizing similarities and interconnectedness with members of the heritage culture group, interdependence may provide a protective cognitive/affective effect from perceptions of intragroup marginalization. Thus, interdependent selves may perceive themselves as more similar to in-group members and as meeting the expectations of the prescribed heritage culture identity. Conversely, because independent selves value autonomy, being unique, and acting in accordance with their own wishes rather than the wishes of an in-group, we surmised that independence may be linked with increased perceptions of intragroup marginalization. Independent individuals may be more likely to perceive themselves as different, for example, by perceiving the mainstream culture as being part of their identity, despite the potential cost of perceiving intragroup marginalization for not remaining similar to other in-group members and maintaining their heritage culture identity. Additionally, by focusing on the distinct and unique aspects of the self, independent selves may feel that they do not conform to the prescribed heritage culture identity, and thus perceive rejection from other heritage culture members. In light of links between perceived rejection and poor socio-emotional functioning (for a review see Wesselmann et al., 2012), the associations of self-construals with intragroup marginalization may, in turn, hold important implications for psychological adjustment and an integrated bicultural identity.

### PSYCHOLOGICAL ADJUSTMENT AND BICULTURAL IDENTITY INTEGRATION

The need to belong is a fundamental human motive, and failure to build lasting social attachments is associated with decreased well-being and adjustment (Baumeister and Leary, 1995; Kahneman and Krueger, 2006). Psychological adjustment is defined as psychological and emotional well-being and is situated in the stress and coping framework (Searle and Ward, 1990). Indeed, intragroup marginalization is linked with increased acculturative stress (Castillo et al., 2008) and decreased subjective well-being (SWB) and flourishing (Ferenczi and Marshall, 2014). We sought to replicate and extend these findings through investigating the associations of intragroup marginalization with two markers of psychological adjustment – SWB and flourishing – and a measure of an integrated bicultural identity.

Subjective well-being, the self-evaluative cognitive component of global life satisfaction (Diener et al., 1985), is a common indicator of psychological adjustment (Chen et al., 2008; Ward and Kus, 2012). SWB is higher in individuals whose personalities match the personality traits that tend to be valued in their heritage culture (Fulmer et al., 2010). Flourishing refers to an individual’s evaluation of their success in five domains: purpose in life, social relationships, self-esteem, self-efficacy, and optimism (Diener et al., 2010). We also measured bicultural identity integration alongside psychological adjustment, which refers to the ways in which individuals perceive their cultural identities as conflicted or compatible, and distant or blended (Benet-Martínez and Haritatos, 2005). An integrated bicultural identity – i.e., when an individual’s cultural identities are harmonious and close – is associated with increased psychological adjustment (Chen et al., 2008).

Previous research indicates that cultures where the interdependent self is prominent tend to be lower in global evaluations of SWB (Diener et al., 1999; Diener and Suh, 2000). However, this may be due to the largely Western conceptualization of the basis of well-being, such as personal goals and consistency (Suh, 2007). Furthermore, the variation of well-being across cultures has been attributed to the link between individualism and increased pursuit of personal goals over social obligations leading to greater happiness (Ahuvia, 2002). By the same token, self-related domains are stronger predictors of SWB for independent individuals. Conversely, relationship-related domains are more important to the SWB of interdependent individuals (Suh et al., 2008; Tam et al., 2010). Indeed, interdependence is linked with greater SWB through relationship harmony, whereas this does not hold for individuals with an independent self-construal (Kwan et al., 1997). Thus, the definitions of well-being, and the pathways to pursue it, may differ depending on which self-construal is dominant. We aimed to extend these findings through incorporating intragroup marginalization as an important intermediary of the associations of self-construal with psychological adjustment and an integrated bicultural identity.

### THE PRESENT RESEARCH

Interdependent and independent self-construals are potentially important predictors of intragroup marginalization. The interdependent self, comprised as it is of its important relationships, roles, and memberships, is sensitive to rejection (Yamaguchi et al., 1995) and values conformity and similarity (Bond and Smith, 1996; Täuber and Sassenberg, 2012). Conversely, the independent self, valuing uniqueness (Kim and Markus, 1999), invests less of the self in any one particular group and values non-conformity (Boucher and Maslach, 2009). We argue that these varying perceptions of similarity or difference with in-group members play a crucial role in whether individuals perceive intragroup marginalization. Thus, individuals primed with interdependence, because they wish to avoid rejection, may see themselves as more similar to other in-group members and perceive less intragroup marginalization. On the other hand, we hypothesized that individuals primed with independent self-construals would perceive themselves as being unique and distinct, and in turn, perceive their heritage culture identity as different and thus rejected by other in-group members. Therefore, we surmised that a primed and chronic interdependent self-construal would be linked with decreased intragroup marginalization. Conversely, a primed and chronic independent self-construal would be linked with increased perceptions of intragroup marginalization. Intragroup marginalization, in turn, would be linked with poor psychological adjustment and a less integrated bicultural identity.

We tested our hypotheses by priming participants (Trafimow et al., 1991). The effects of priming self-construal tend to mirror cultural differences in chronic self-construal, regardless of individuals’ cultural origins (Gardner et al., 1999). We also assessed chronic self-construals through a self-report measure. Testing both primed and chronic self-construal allowed us to more accurately discern the association of self-construals with perceived intragroup marginalization.

## MATERIALS AND METHODS

### PARTICIPANTS

Two hundred and seventy-eight participants (*M*_age_: 28.53, SD: 8.52; female: 121, male: 157) completed the questionnaire. As the experiences of intragroup marginalization as a general construct were of focal interest, sampling was conducted for a variety of heritage cultures. Participation criteria required participants to have a heritage culture that was different to their mainstream culture. The majority of participants reported a European heritage culture (25%), followed by South American (21%), East Asian (14%), African/Caribbean (12%), South Asian (9%), Mixed (7%), Southeast Asian (6%), North American (3%), Middle Eastern/North African (1%), Jewish (1%), or Australian/New Zealand (1%). Classification of heritage cultures in terms of Hofstede’s (2001) ratings of individualism revealed that 208 (75%) participants reported a heritage culture that is low in individualism, and 70 (25%) a culture high in individualism. Regarding the mainstream culture, participants were given the definition of a mainstream culture being the culture that they had moved to or were born in, that was *different* to their heritage culture. The majority of participants reported living in North America (82%). They also reported the following mainstream cultures: European (15%), Asian (1%), Middle Eastern/North African (1%), or South American (1%). As the majority of the mainstream cultures were classified as high in individualism (97%), this variable was not included in the analyses.

One hundred and thirty-three (48%) participants reported that they were first-generation migrants (*M*_years residing in host culture_: 10.62, SD: 8.38), and 145 (52%) participants reported that they were second/later generation migrants or bicultural individuals. The majority of participants were working full-time or were currently at university (combined 77%). Participants were highly educated with the majority reporting at least having completed or were pursuing an undergraduate degree (89%). Participants were recruited via Amazon MTurk, with a payment of 35 cents USD for completion of the questionnaire; this method of recruitment has been shown to be as reliable as other collection procedures, with the added benefit of providing a more diverse and representative sample of the general population in contrast to traditional university student samples (Buhrmester et al., 2011; Casler et al., 2013).

### MATERIALS AND PROCEDURE

Ethics approval was received from the Ethics Committee of the Department of Psychology at Brunel University, in accordance with the recommendations of the British Psychological Society. Participants provided informed consent, and were given the opportunity to contact the researchers, refuse to participate, or withdraw at any time without consequences. Participants first completed the socio-demographic questions and a measure of chronic self-construal (Singelis, 1994). They were then randomly assigned to one of three self-construal prime conditions: interdependent self (*N* = 92), independent self (*N* = 83), and control (*N* = 103). After the priming task, participants completed a manipulation check and measures of intragroup marginalization, SWB, flourishing, and bicultural identity integration. All materials were in English.

#### Self-Construal Scale (Singelis, 1994)

Seven items from the Self-Construal Scale were included to measure independence, and eight items to measure interdependence. Participants indicated the extent of their agreement on a 7-point Likert scale (1 = *Not true at all*, 7 = *Very True*). Because we used a short-form of this scale, we analyzed its structure using principal axis factoring with varimax rotation, following Singelis’s (1994) finding that the two self-construal dimensions are orthogonal. We expected only two factors to emerge, but found that three factors had eigenvalues over 1, which accounted for 33.29% of the total variance. Only the two most dominant factors were clearly interpretable, and corresponded with interdependence and independence. Five items loaded onto the first factor, which represented interdependence (e.g., “I have respect for the authority figures with whom I interact”; α = 0.64). One cross-loading item was removed. Three items loaded onto the second factor, which represented independence (e.g., “Speaking up during class is not a problem for me”; α = 0.62). The remaining items did not load sufficiently strongly on any factor, they were cross-loaded, or they loaded weakly on the third, uninterpretable factor. Because of its somewhat anomalous factor structure, we were cautious in our interpretation of any results based on this scale.

#### Self-construal prime (Trafimow et al., 1991)

Self-construal was manipulated using a task that makes salient either an interdependent or independent self. Participants were asked to reflect on either the similarities (interdependent) or the differences (independent) that they may have with their family and friends. In addition, they were asked to reflect on either what others might expect of them (interdependent), or conversely, on their own self-expectations (independent). Manipulating the accessibility of either self or others’ expectations is an important aspect in which the two self-construals differ. Research by Trafimow et al. (1991) has demonstrated that independent and interdependent self-cognitions are distinct and separate, and that priming one increases the ease with which pertinent information regarding that aspect of the self is retrieved. Participants were shown one of three primes and asked to spend 3 min writing a response: (1) What they had in common with their family and friends and what they felt their family and friends expected of them (interdependent self); (2) What made them different to their family and friends and what their own expectations were of themselves in general (independent self); (3) The route, in detail, that they took daily to their university or place of employment to engage their imagination without priming self-construal (control condition). We did not specify that participants refer to heritage culture family and friends in their responses; although family members were most likely from the heritage culture, friends could be from any culture, thus allowing individuals to consider similarities and differences from their chosen friends.

#### Manipulation check

A manipulation check question asked participants to indicate the extent of their agreement with the statement “It is important for me to maintain harmony with my group” on a 5-point Likert scale (1 = *Strongly Disagree,* 5 = *Strongly Agree*). In addition, in order to provide further evidence that the manipulation had the intended effect, the responses to the open-ended primes were coded for themes reflecting independence, interdependence, or neither.

#### Intragroup Marginalization Inventory (IMI; Castillo et al., 2007)

Two subscales of the Intragroup Marginalization Inventory (IMI), developed to capture the perceptions of intragroup marginalization by members of an individual’s heritage culture, were used in the present study. The family subscale (e.g., “Family members criticize me because I don’t speak my heritage/ethnic group’s language well”) centers on experiences of rejection from family due to acculturating and adopting the mainstream culture in ways that are deemed as a threat to the heritage culture social identity (11 items; α = 0.80). The friends subscale (e.g., “Friends of my heritage culture group tell me that I have too many friends from the mainstream culture”) focuses on experiences of rejection from friends who are from the heritage culture (16 items; α = 0.91). Participants indicated the extent to which the items occurred in their daily lives on a 7-point Likert scale (1 = *Never/Does not apply*, 7 = *Extremely Often*).

#### Satisfaction with Life Scale (SWLS; Diener et al., 1985)

The Satisfaction with Life Scale (SWLS) is composed of five statements that capture overall satisfaction with one’s life (α = 0.91; e.g., “So far I have gotten the important things in my life”). Participants indicated on a 5-point Likert scale (1 = *Strongly Disagree,* 5 = *Strongly Agree*) the extent of their agreement.

#### Flourishing Scale (Diener et al., 2010)

Flourishing (eight items; α = 0.93) was included as an additional measure of psychological adjustment (e.g., “I am competent and capable in the activities that are important to me”). Participants indicated on a 7-point Likert scale (1 = *Strongly Disagree,* 7 = *Strongly Agree*) the extent of their agreement.

#### Bicultural Identity Integration Scale (BIIS-1; Benet-Martínez and Haritatos, 2005)

The Bicultural Identity Integration Scale (BIIS-1) is composed of two subscales with four items each. Cultural identity distance measures the perceived distance between one’s heritage and mainstream culture identities (α = 0.66; “I am simply a migrant/member of an ethnic/heritage culture group who lives in a host/mainstream culture”). Cultural identity conflict captures the perceived conflicts that arise from holding both heritage and mainstream culture identities (α = 0.76; “I feel caught between my ethnic/heritage and host/mainstream cultures”). Participants indicated on a 5-point Likert scale (1 = *Disagree Strongly*, 5 = *Agree Strongly*) the extent of their agreement with each of the items, with higher scores representing higher levels of cultural distance and conflict.

## RESULTS

### MANIPULATION CHECK

We conducted a one-way ANOVA on the manipulation check item, but it was not significant, *F*(2,274) = 1.53, *p* = 0.22. However, *post hoc* tests revealed that the difference between responses to the independent (*M*: 3.70, SD: 0.95) and interdependent (*M*: 3.95, SD: 0.92) primes approached significance, *t*(173) = 1.75, *p* = 0.08, and was in the predicted direction. Responses to the neutral prime lay between the two other groups (*M*: 3.83, SD: 0.94). In addition, two coders blind to condition assessed participants’ open-ended responses to the priming tasks. They coded for the following features: similarities to others and/or expectations that others might have of the participant (interdependent self-construal), description of uniqueness or distinctiveness of self and/or self-expectations (independent self-construal), or no mention of either (neither/neutral). Inter-rater agreement, κ = 0.90, [CI: 0.85, 0.94], was near perfect (Landis and Koch, 1977). The coders, still blind to condition, then discussed those cases where there had been a discrepancy and came to an agreement, which formed the combined coder score. The agreement between the combined coder score and the actual condition that participants had been assigned to was also near perfect, κ = 0.85, [CI: 0.82, 0.88]. Buttressing the results for the manipulation check item, then, these findings suggest that the primes were successful in activating independent, interdependent, or neutral schemata.

### SELF-CONSTRUAL AND INTRAGROUP MARGINALIZATION

Pearson’s correlation coefficient, mean, and standard deviation are reported in **Table 1**. We tested the effect of self-construal priming on family and friend intragroup marginalization with two hierarchical regression models. All continuous variables were centered on the grand mean. The following variables were entered in the first step: age; individualism of the participant’s heritage culture, based on Hofstede’s (2001) ratings of individualism at the national level (effect coded as –1 for a culture high in individualism and 1 for a heritage culture low in individualism); and generational status (effect coded as –1 for a second-generation+/bicultural individual, and 1 for a first-generation migrant). Priming condition was included in the second step. Two contrasts were created: one which contrasted the interdependent condition with the control condition (–1 = control; 1 = interdependent; 0 = independent) and is referred to as the interdependent condition variable, and another which compared the independent condition to the control condition (–1 = control; 1 = independent; 0 = interdependent) and is referred to as the independent condition variable.

**Table 1:** Mean, standard deviation, and Pearson correlation for variables.

Variable	1	2	3	4	5	6	7
(1) Age							
(2) Family IGM	–0.15*						
(3) Friend IGM	–0.13*	0.69**					
(4) SWB	0.02	–0.19**	–0.01				
(5) Flourishing	0.10	–0.30**	–0.21**	0.76**			
(6) Bicultural distance	–0.14*	0.22**	0.21**	–0.08	–0.19**		
(7) Bicultural conflict	–0.03	0.44**	0.37**	–0.16**	–0.16**	16**	
*Grand mean (SD)*	28.49 (8.48)	36.36 (12.15)	47.68 (18.65)	16.81 (5.24)	42.23 (9.66)	10.71 (2.96)	11.02 (3.97)
*Neutral condition mean (SD)*	28.93 (8.55)	36.16 (13.04)	45.74 (18.49)	16.66 (5.68)	42.39 (10.04)	10.72 (3.17)	10.89 (3.96)
*Interdependent condition mean (SD)*	28.22 (8.64)	34.44 (12.04)	49.26 (19.85)	17.38 (4.83)	43.18 (9.05)	10.67 (2.67)	10.90 (4.00)
*Independent condition mean (SD)*	28.22 (8.40)	38.77 (10.82)	48.24 (17.41)	16.36 (5.19)	41.07 (9.78)	10.64 (3.06)	11.35 (4.01)

*p < 0.05, **p < 0.01.

There were significant main effects of both contrasts on family but not friend intragroup marginalization (**Table 2**). Relative to participants in the control condition, participants primed with interdependence reported lower family intragroup marginalization, and participants primed with independence reported higher family intragroup marginalization. Supporting our hypothesis, these results indicated that priming an interdependent self-construal through emphasizing similarity with other in-group members provided a protective effect against perceived intragroup marginalization. Conversely, priming an independent self-construal through emphasizing uniqueness and difference to others increased family intragroup marginalization. There were no significant interactions between the levels of individualism of participants’ heritage cultures and primed self-construal.

**Table 2:** Predictors of family and friend intragroup marginalization.

Model	Family IGM		Friends IGM	
	Unstandardized β	Standardized β	Unstandardized β	Standardized β
Step 1/constant	42.35		55.15	
Age	–0.22	–0.15*	–0.28	–0.13*
Heritage culture individualism	–0.02	–0.00	0.50	0.02
Generational status	–0.40	–0.03	–0.69	–0.04
Step 2/constant	42.58		55.06	
Age	–0.22	–0.15*	–0.27	–0.12*
Heritage culture individualism	–0.17	–0.01	0.58	0.03
Generational status	–0.39	–0.03	–0.59	–0.03
Condition: interdependent	–2.03	–0.14*	1.57	0.07
Condition: independent	2.38	0.16*	0.57	0.03

*p ≤ 0.05.

We then investigated the two factors derived from factor analysis that represented chronic interdependence and independence. Bivariate correlations of the chronic interdependence factor with family and friend intragroup marginalization mirrored the associations found with primed interdependence. Thus, chronic interdependence was linked with decreased perceptions of intragroup marginalization from family, *r* = –0.14, *p* < 0.05, and friends, *r* = –0.20, *p* < 0.05, further bolstering our priming results. Chronic independence was not correlated with intragroup marginalization. We included these two factors indexing chronic interdependence and independence in our regression models predicting family and friend intragroup marginalization. The priming effects of interdependence, β = –0.15, *p* < 0.05, and independence, β = 0.16, *p* < 0.05, remained significant in predicting family intragroup marginalization. Additionally, chronic interdependence was linked with decreased friend intragroup marginalization, β = –0.19, *p* < 0.005; it was not significantly associated with family intragroup marginalization. While the results for chronic interdependence are in line with the results for primed interdependence, the anomalous nature of the measure of chronic self-construal led us to focus on primed self-construal for the remaining analyses.

### INDIRECT EFFECTS OF SELF-CONSTRUAL ON PSYCHOLOGICAL ADJUSTMENT VIA IGM

Hierarchical regression models indicated that family intragroup marginalization was significantly correlated with both indicators of psychological adjustment, and bicultural identity conflict (**Table 3**), providing support for indirect effects of self-construal on psychological adjustment and identity conflict through family intragroup marginalization (Baron and Kenny, 1986). Bootstrap procedures (Preacher and Hayes, 2008) then tested whether there were any indirect effects of independent and interdependent self-construals on psychological adjustment and identity conflict through intragroup marginalization. Six mediation models were tested. To replicate the hierarchical regression models, all previous control variables were included as covariates (age, generational status, and heritage culture individualism), along with friend intragroup marginalization, and the opposing priming condition contrast variable. Indirect effects are tested by examining the significance of four pathways: the association of the independent variable with the mediating variable through which the indirect effect is exerted (a-path), the association of the mediating variable with the outcome variable (b-path; the combination of the a and b paths representing the indirect effects), the total effect (c-path), which measures the complete association of the predictor and outcome variable, and the direct effect (c’-path), which accounts for the association between the predictor and outcome variables when controlling for the indirect effects.

**Table 3:** Association of intragroup marginalization with psychological adjustment and bicultural identity integration.

Model	SWB		Flourishing		Bicultural identity conflict		Bicultural identity distance	
	Unstandardized β	β	Unstandardized β	β	Unstandardized β	β	Unstandardized β	β
Step 1/constant	16.99		39.01		11.41		11.48	
Age	0.01	0.01	0.12	0.10	–0.02	–0.04	–0.03	–0.10
Heritage culture individualism	–0.43	–0.08	0.24	0.02	0.30	0.07	0.32	0.10
Generational status	0.65	0.12*	0.07	0.01	0.13	0.03	0.55	0.19**
Step 2/constant	19.17		48.75		5.01		9.17	
Age	–0.00	–0.00	0.07	0.06	0.02	0.03	–0.02	–0.06
Heritage culture individualism	–0.48	–0.08	0.16	0.01	0.39	0.90	0.33	0.10
Generational status	0.62	0.12	–0.10	–0.01	0.24	0.06	0.59	0.20**
Condition: interdependence	0.18	0.03	0.57	0.05	0.23	0.05	0.13	0.04
Condition: independence	–0.13	–0.02	–0.72	–0.06	0.02	0.00	–0.15	–0.04
Family intragroup marginalization	–0.13	–0.29**	–0.22	–0.27**	0.12	0.37**	0.02	0.09
Friend intragroup marginalization	0.06	0.20*	–0.01	–0.02	0.02	0.09	0.02	0.15

*p ≤ 0.05, **p ≤ 0.005.

**FIGURE 1:**
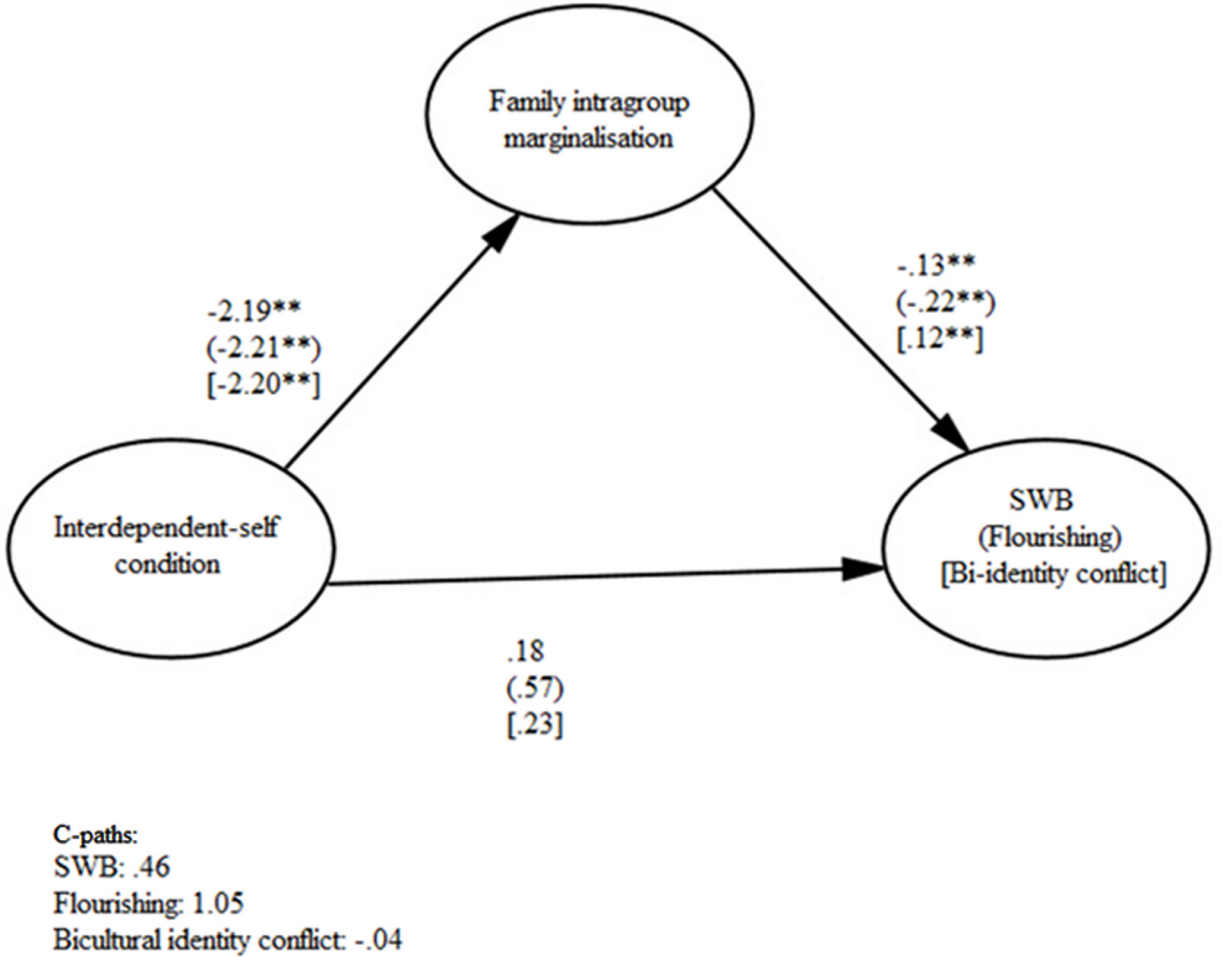
**The indirect effects of primed interdependent self-construal on SWB, flourishing, and bicultural identity conflict via family intragroup marginalization**.

Examination of the 95% bias-corrected confidence intervals (CIs) from 5,000 bootstrap samples revealed support for all six indirect effects of primed self-construal on psychological adjustment and identity conflict through family intragroup marginalization. The indirect effects of primed interdependent self-construal via family intragroup marginalization on SWB [CI: 0.08, 0.64], flourishing [CI: 0.13, 1.07], and bicultural identity conflict [CI: –0.52, –0.09] were significant (all three pathways are illustrated in **Figure 1**). Priming interdependent self-construal therefore appeared to be linked with decreased intragroup marginalization and, in turn, its detrimental effect on psychological adjustment and identity conflict.

**FIGURE 2:**
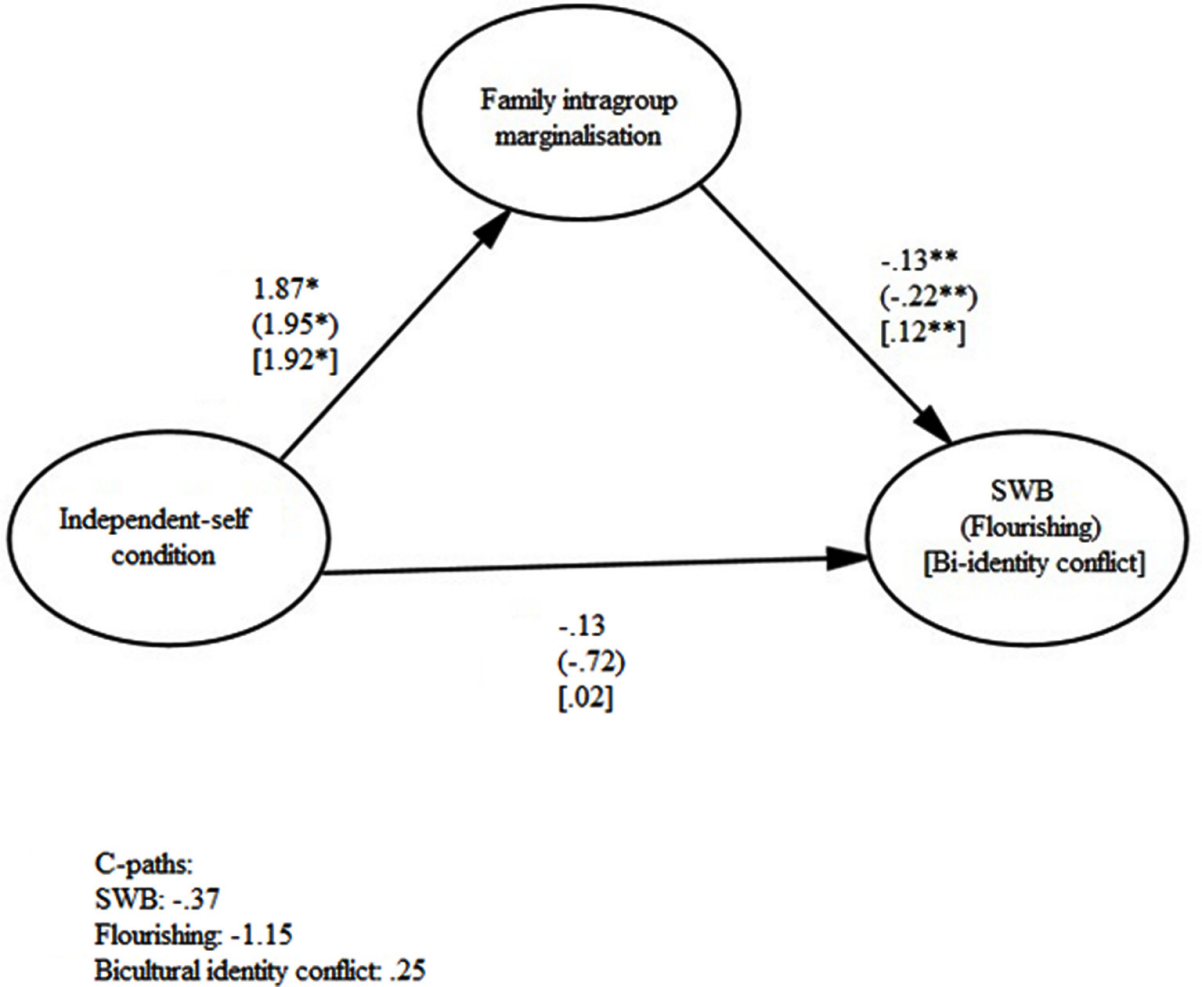
**The indirect effects of primed independent self-construal on SWB, flourishing, and bicultural identity conflict via family intragroup marginalization**.

The indirect effects of primed independent self-construal through increased intragroup family marginalization on the two indicators of psychological adjustment and identity conflict were also significant: the pathways for SWB [CI: –0.56, –0.05], flourishing [CI: –1.06, –0.09], and bicultural identity conflict [CI: 0.05, 0.50] are depicted in **Figure 2**. Thus, participants primed with an independent self-construal, relative to those in the control condition, reported increased family intragroup marginalization, which in turn was associated with decreased psychological adjustment and increased identity conflict. For each of the six models, there was a decrease between the total effect (c-path) and the direct effect (c’-path), indicating partial mediation through family intragroup marginalization, although neither of the paths were significant. The lack of a significant c-path does not disconfirm a partial indirect effect via a mediating variable, particularly when the causal process between the predictor and outcome variables is complex and lateral (Shrout and Bolger, 2002). Indeed, a significant indirect effect of *X* on *Y* through *M* (the mediating variable) is valid despite the lack of a significant association between *X* and *Y*; the total association path, after all, theoretically includes all of the direct and indirect paths between the two variables, which may act in opposing directions and are not all measured in the proposed model (Hayes, 2009). The six mediation models support the presence of a pathway between self-construal to psychological adjustment and identity conflict through family intragroup marginalization.

## DISCUSSION

Collectively, our results supported our hypothesis that priming an interdependent self-construal by emphasizing similarity to others exerted a protective effect on psychological adjustment (SWB and flourishing) and bicultural identity conflict through decreased perceptions of intragroup marginalization from family. Conversely, priming an independent, unique self resulted in increased perceptions of family intragroup marginalization, which in turn was linked with poor psychological adjustment and increased identity conflict. This research provides further insight into the differing pathways linking self-construal with psychological adjustment and identity conflict, specifically, in the beneficial aspects of interdependent self-construal.

### PRIMED SELF-CONSTRUAL AND INTRAGROUP MARGINALIZATION

Priming the interdependent self was linked with decreased perceived family intragroup marginalization relative to a neutral prime. Notably, the interdependent self-construal prime asked participants to recall the ways in which they were similar to their family and friends and the expectations that they perceived were required of them by others. Making these expectations salient decreased perceptions of rejection. We surmise that through asking participants to reflect on the similarities between themselves and close others in the priming task, less information about discrepancies between the self and others is accessible when recalling experiences of intragroup marginalization. In line with previous findings that private and collective self-cognitions are stored in separate locations in memory (Trafimow et al., 1991; Singelis, 1994), our results imply that perceived intragroup marginalization taps into the discrepancy between cognitions of the private and collective self, particularly in reference to the tensions and expectations of the heritage culture social identity. Furthermore, self-consistency is only weakly linked with well-being and perceptions of authenticity for individuals with a relational-interdependent self (Cross et al., 2003). The priority is thus shifted to fitting in successfully within different contexts (Markus and Kitayama, 1991, 1994; Cross et al., 2003). When primed with an interdependent self, individuals are thus much more aware of the standards and expectations of heritage culture members on their social identity. Consequently, individuals may perceive acceptance from their heritage culture without experiencing compromise or inauthenticity for their self.

Conversely, participants primed with an independent self-construal reported increased perceived family intragroup marginalization relative to those in the neutral condition. We argue that by requiring individuals to reflect on what they expect of themselves and the ways that they differ from their family and friends primes the independent self, and in this vein, the notion that the self is separate and unique from others (Markus and Kitayama, 1991). One of the links to increased intragroup marginalization may be the focus on the private self at the expense of the collective self during recall (Trafimow et al., 1991). The Intragroup Marginalization Inventory (Castillo et al., 2007) centers on the premise of difference, an attribute that is part and parcel of the independent self. Thus, individuals primed with independence attend to those instances in which their attributes differed from the prescribed heritage culture identity that they perceived their family expected of them. The link between consistency and authenticity has been reported to be highly significant for individuals low in relational self-construal (Cross et al., 2003); for independent individuals it may be crucial to maintain the authenticity of their self through consistency, rather than prescribing to the heritage culture identity when interacting with other in-group members. The ideal self for individuals with independent self-construal is composed of autonomous self-knowledge (e.g., traits, attitudes, preferences) and is context independent (Hannover et al., 2006). These findings provide further support that individuals primed with independent self-construal recall experiences of behaving in line with their inner self at the expense of acceptance by the heritage culture. Further research could seek to establish whether individuals who feel rejected by heritage culture members derogate them in response (Bourgeois and Leary, 2001). Taken together, our results imply that interdependent self-construal serves a protective function against perceived intragroup marginalization, whilst independent self-construal increases perceived rejection from members of the heritage culture.

In contrast to the significant effects of primed self-construal, only chronic interdependence was linked with decreased intragroup marginalization from friends; however, bivariate correlations indicated a similar pattern for family intragroup marginalization. Although these results bolster our theoretical arguments, we hesitate to over-interpret the results because we used a non-standard measure of self-construal. However, these results do suggest that chronic and primed self-construal show similar associations with intragroup marginalization. We surmise that this lack of association may be owing to the scale we used to measure chronic self-construal. Future research should seek to replicate the results for chronic self-construal using the full version of the Self-Construal Scale. It should be noted that prior meta-analytic research on the construct validity of the Self-Construal Scale, along with two other measures of chronic self-construal, has demonstrated its inconsistency (Levine et al., 2003a,b). Researchers have questioned the presence of a Western bias evidenced in the use of self-report measures which are incompatible with the flexible nature of an interdependent self-construal (Markus and Kitayama, 1998), the suitability of a two-factor structure of self-construal as the best fit to the data (Hardin et al., 2004), and whether the Self-Construal Scale measures that which it purports (Levine et al., 2003a). Further research should investigate the relationship between chronic self-construal and perceived intragroup marginalization through using different measures of self-construal (e.g., Gudykunst et al., 1996). Nonetheless, the significant effects of the priming measure indicate that self-construal has an impact on perceptions of intragroup marginalization.

It is important to note that priming self-construal affected perceptions of marginalization from family only; perceptions of marginalization from friends were not affected. Moreover, only family intragroup marginalization was associated with decreased psychological adjustment. This pattern of results may be explained by two reasons. First, we did not assess the number of heritage and mainstream culture friends that participants had. Because of the voluntary nature of friendships (Hays, 1988), if individuals perceive rejection from heritage culture friends, they may choose to leave those friendships and form new ones. Such a reaction toward family members is not as easily available, as family ties may be perceived as less controllable and more permanent than friendships. Thus, it is possible that some participants did not have enough heritage culture friends from whom they felt marginalized. Second, it may be that friendships do not exert the same impact on well-being as family relationships do. Chronic perceptions of rejection from one’s family may be seen as a relatively irremediable; whereas individuals can leave or deprioritise friendships where they feel rejected, they may feel bound to their family, and, in turn, experience poor psychological adjustment and a conflicted bicultural identity.

### INDIRECT EFFECTS OF PRIMED SELF-CONSTRUAL ON PSYCHOLOGICAL ADJUSTMENT AND BICULTURAL IDENTITY CONFLICT VIA IGM

The positive and negative effects of interdependent and independent primed self-construal, respectively, carried over through both indicators of psychological adjustment and identity conflict through family intragroup marginalization. Priming individuals with an interdependent self decreased perceptions of family intragroup marginalization, which, in turn, was linked with decreased SWB and flourishing. Previous research links interdependent self-construal with decreased personal well-being (Diener et al., 1999; Diener and Suh, 2000); for example, Suh (2007) highlights the disadvantages of an interdependent self-construal when approached from a Western perspective of well-being because aspects of an interdependent self are incompatible with the pursuit of personal well-being. In contrast, the current findings suggest that the interdependent self enhances psychological adjustment. Our results provide further support for the distinction between the motivations and methods that individuals with differing self-construals pursue in attaining psychological well-being. In regards to bicultural identity conflict, the results suggest that perceiving the self as similar and embedded in one’s social relationships exerts a protective effect over intragroup marginalization. In turn, the perceived external pressure of intragroup marginalization increases conflict between one’s mainstream and heritage culture identity. We surmise that one important aspect of a harmonious bicultural identity is the perception that one’s identity is accepted and valued by other members of the heritage culture. The pathway between self-construal and psychological adjustment is likely complex as there were no significant direct effects between the two. Further research should seek to replicate the beneficial effects of an interdependent self on psychological adjustment and decreased identity conflict through an increased sense of acceptance by one’s heritage culture.

In contrast, individuals primed with an independent self-construal reported increased perceived family marginalization, which in turn was associated with decreased SWB and flourishing. Regarding bicultural identity conflict, priming independence increased perceptions of rejection from family, and in turn, a conflicted bicultural identity. Making salient to individuals the ways that they are different from close others may account for the detrimental indirect effects on psychological adjustment and increased identity conflict. The sense of belonging is a basic human need (e.g., Maslow, 1968; Baumeister and Leary, 1995), even for independent individuals. The current findings provide support for the “dark sides of each culture” (Suh, 2007, p. 1338), through portraying one of the pathways in which independent self-construal has an indirect effect on poor psychological adjustment and increased identity conflict. Further research should investigate whether individuals primed with an independent self-construal reap benefits from other areas of their life, such as through identification with the mainstream culture, or from convictions of authenticity and self-consistency (Cross et al., 2003) in the face of intragroup marginalization.

### LIMITATIONS AND FURTHER RESEARCH

The limitations of the present study center on inclusion of further variables and the participant sample demographics. First, we did not measure social interaction with members of the mainstream culture. There may exist yet uncovered links between primed self-construal and intragroup marginalization that are mediated by the degree of interaction and affiliation with the mainstream culture, as exemplified by the link between interdependent self-construals and positive interactions with members of the mainstream culture (Nezlek et al., 2011). In terms of outcome variables, further research could extend the present findings through the inclusion of other indicators of adjustment, including acculturative stress (Benet-Martinez, 2003; Miller et al., 2011), depression, negative emotions, and physical health. Future research can also seek to investigate whether self-construal predicts intragroup marginalization, and, in turn, a conflicted bicultural identity. Does a conflicted bicultural identity in turn predict decreased psychological adjustment? Although the present sample size was too small to test this multiple-mediator model with structural equation modeling, further research can address this limitation.

We recruited a cross-cultural participant sample to investigate the link between self-construal and intragroup marginalization. Due to the lack of geographical constraint in participant collection, the distribution of participants’ heritage cultures on the individualism spectrum was unequal, with most falling on the low-individualist end. Additionally, due to the extensive variety of the participants’ heritage and mainstream cultures, hierarchical linear modeling was not possible. However, we controlled for individualism levels of heritage culture by including the effect coded variable in the analyses, which did not influence the pattern of findings. If anything, the present findings attest to the cross-cultural resonance of perceptions of rejection from one’s heritage culture.

Finally, we focused on only one aspect of interdependence and independence – similarity to or difference with close others. Indeed, the priming measure by Trafimow et al. (1991) operationalized interdependence as similarity to close others, and independence as uniqueness from close others. Further research should seek to replicate our findings by priming other aspects of an interdependent and independent self-construal, such as obligation to one’s in-group versus following one’s own wishes. Such research would further clarify whether certain aspects of the interdependent self – perceived similarity to the in-group versus feeling obligated – provide a protective effect against perceptions of intragroup marginalization and the resulting poor psychological adjustment.

## CONCLUSION

Our results showed that priming an interdependent self – in particular, perceived similarity with family and friends – protected individuals from the detrimental effects of perceived intragroup marginalization on psychological adjustment and identity conflict. Conversely, priming the unique characteristics of an independent self increased perceptions of intragroup marginalization and, in turn, decreased psychological adjustment and increased identity conflict. The interdependent self may exert beneficial effects through focusing on similarities with other members of the heritage culture, whereas, the independent self may increase perceptions of intragroup marginalization through focusing on the ways that one is unique to other members of their heritage group during recall of intragroup marginalization. Clinical interventions that focus on the similarities between oneself and other heritage culture members, and the importance of feeling that one lives up the expectations of their in-group may provide respite from perceived intragroup marginalization. In turn, this may promote well-being, flourishing, and a harmonious bicultural identity. Our findings suggest that individuals are not islands, separate and free from the restrictions of their heritage culture; maintaining separation carries repercussions for well-being.

## Conflict of Interest Statement

The authors declare that the research was conducted in the absence of any commercial or financial relationships that could be construed as a potential conflict of interest.
